# Temporal dynamics of gut biosynthetic gene clusters link persistent colonization and engraftment in fecal microbiota transplantation

**DOI:** 10.1080/19490976.2026.2634469

**Published:** 2026-02-25

**Authors:** Fernando Garcia-Guevara, Tom Resink, Frederick Clasen, Mathias Uhlén, Adnane Achour, Saeed Shoaie

**Affiliations:** a Centre for Host-Microbiome Interactions, Faculty of Dentistry, Oral & Craniofacial Sciences, King's College London, London, UK; b Department of Medicine Science for Life Laboratory, Karolinska Institute, Solna, Sweden; c Division of Infectious Diseases, Karolinska University Hospital, Stockholm, Sweden; d Department of Protein Science, Science for Life Laboratory, KTH-Royal Institute of Technology, Stockholm, Sweden; e Quantitative Systems Biology, Faculty of Medicine, Biruni University, Istanbul, Türkiye

**Keywords:** Biosynthetic gene clusters, secondary metabolites, human microbiota, fecal microbiota transplantation

## Abstract

The human gut microbiome carries a large array of biosynthetic gene clusters (BGCs) that encode the production of secondary metabolites, yet their temporal dynamics and role during microbial colonization remain largely unexplored. Here, we tracked BGCs profile over time in a cohort of healthy adults, and identified two distinct groups: persistent, which are stable over time, and transient, which are more sporadic. Functional annotations indicated persistent gene clusters are enriched in antibiotic resistance mechanisms, while transient ones more frequently carry virulence-associated genes. We then examined colonization of these two groups in the context of fecal microbiome transplantation. Our results show that persistent gene clusters exhibit higher colonization rates than transient ones. These findings contribute to our understanding of how microbial metabolites influence host health, potentially guiding future therapeutic strategies targeting the microbiome.

## Introduction

Bacterial secondary metabolites (SM) mediate interactions between microbial species, their host, and environmental changes. Although SM is not essential for bacterial growth, it represents a major source of bioactive molecules such as antibiotics.^1^ SM can shape microbial ecosystems, modulating the microbiome niche differentiation and adaptation in complex environments.^2^ The potential of a bacterial SM can be predicted based on specific genome features known as biosynthetic gene clusters (BGCs), as the enzymes involved in their synthesis are typically encoded near each other in the genome sequence. Apart from detecting conserved protein domains associated with SM, deep learning-based genome mining and the integration of pan-genomics have extended BGC class characterization including divergent or previously uncharacterized BGCs.^3-11^ Additionally, incorporating features such as evidence of horizontal gene transfer or the presence of antibiotic resistance genes improves both the prediction of BGCs and the prioritization of candidate products for functional characterization.^12^ SM is enriched in the human microbiome, and mediate host-microbiome interactions, including immune modulation, antimicrobial activity, genotoxic effects, and nutrient scavenging.^13^ For instance, lactocillin, encoded by a BGC in *Lactobacillus gasseri*, displays antimicrobial activity against a specific range of pathogens.^14^ Colibactin, the product of a BGC in *Escherichia coli*, has been linked to its virulence, shown to induce DNA damage^15^ and to be associated with colorectal cancer.^16^ Previous studies have highlighted the rich biosynthetic potential of the human microbiome,^14^^,^^17^ however, little is known about the temporal dynamics of BGCs and how microbiome interventions such as fecal microbiota transplantation (FMT) affect BGC composition also the how BGC composition can affect the successful engraftment, and how this composition in turn influences successful engraftment.

The distribution of gut bacterial species in healthy individuals can undergo specific transitions over time, with microbes possessing persistent or transient colonizing properties, that affect the overall microbial phenotypes.^18^ Since BGCs form a significant part of the metabolic repertoire of these microbial species, BGCs themselves may display analogous patterns of persistence and transience. In-depth biological annotations could provide valuable insights into the ecological roles of BGCs in the gut and improve the functional interpretation of predicted clusters.^12^ Knowledge of the temporal dynamics of BGCs allows a better understanding of how SM contribute to the microbial composition structure. BGC content alone can alter SM profiles observed in simple co-culture experiments,^19^ which are commonly employed to activate the expression of cryptic biosynthetic genes.^20^ In this context, FMT can be considered a large-scale coculture experiment. During microbial colonization, donor-derived microbes are introduced into a new environment, where SM may play a central role in establishing their niche. Metagenomic datasets from FMT studies offer a valuable opportunity to track BGCs during colonization events, and to investigate their contribution to shaping the recipient microbiome.

In this study, we aimed to comprehensively describe the temporal dynamics of human gut microbiome BGCs using predictive modeling and assess the presence of these BGCs in FMT trial cohorts to elucidate their potential roles. We generated a metagenome-based BGC catalog using longitudinal data from a healthy population. We described BGC profiles over time, revealed the dynamics of the BGC contents under healthy conditions, and identified transient and persistent groups of BGCs, setting a potential reference for common and rare BGCs in the human gut. The two groups presented different functional properties in terms of antibiotic resistance capacity and virulence. We thereafter tracked BGCs transferred from healthy donors into patients during colonization events in FMT treatments across different trials, under the perspective of transient and persistent BGCs. By linking longitudinal BGC dynamics to colonization success and functional annotation, our work provides a foundation for prioritizing candidate BGCs for downstream characterization, with potential implications in microbiome-targeted and live bacterial therapeutics.

## Results

### Construction of the biosynthetic gene cluster catalog from longitudinal healthy gut microbiome samples

**Figure 1.**
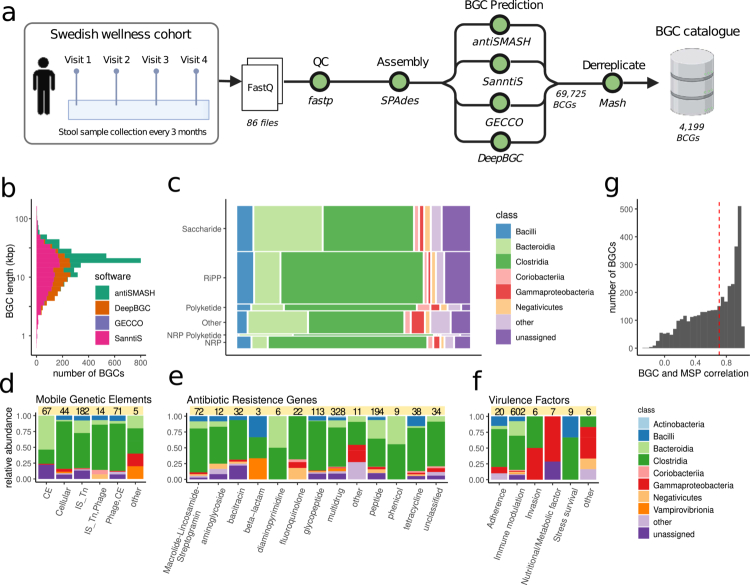
Creation of a BGC wellness catalog. a) The scheme shows the pipeline for building the BGC catalog employed in this study. First, a BGC catalog in healthy individuals was constructed using the Swedish wellness cohort. In this study, gut microbiome samples were collected from a group of 86 healthy adults every three months during a period of 1-y. The shotgun metagenomic samples were analyzed with the Nextflow BGC prediction pipeline (see methods). b) Histogram nucleotide length of each predicted BGC in the catalog colored by the prediction tool. c) Mosaic plot of BGC type and taxonomic Class. The rectangle area is proportional to the relative abundances of the taxonomy annotations at Class level and Product types of the predicted BGC. Bar-plots show the relative abundance of the types of d) mobile genetic elements (CE, conjugative elements; IS, insertion sequences; Tn, transposons), e) antibiotic resistance genes, and f) virulence factor categories identified in the BGC catalog colored by taxonomic class. Integer values on top of plots d, e, and f show the number of BGC found within each category. g) Histogram of Spearman correlation values between BGC abundance and the MSP abundance with the same taxonomic annotation as the BGC.

To construct a comprehensive *de novo*-assembled BGC catalog, we analyzed shotgun metagenomic data from 86 healthy individuals from Sweden, sampled longitudinally for 4 times over 1 y (hereafter referred to as the Wellness cohort; Figure 1a).^18^^,^^21^ Raw metagenomic reads from the Wellness cohort were assembled into contigs using SPAdes^22^ after which we employed four established algorithms (antiSMASH, SanntiS, GECCO, and DeepBGC)^5^^,^^8-10^ to predict BGCs from these assemblies (**Methods**). Initially, 69,725 BGCs were identified, of which 4,199 unique representative BGCs remained after redundancy removal. The median length of these representative BGCs was approximately 17.5 kbp (Figure 1b), with the longest sequences predicted by antiSMASH.^10^ Among all identified BGC types, saccharides were the most abundant, followed by ribosomal synthesized and post-translational modified peptides (RiPPs) and non-ribosomal peptide synthetases (NRPS). Taxonomically, BGCs from the class Clostridia were most prevalent, followed by those from Bacteroides and Firmicutes (Figure 1c).

To assess the presence, absence, and relative abundance of each representative BGC across samples, we mapped shotgun metagenomic reads from each individual in the Wellness cohort against the representative BGC catalog. On average, 880 BGCs (±163 SD) were detected per individual across all the sampling points. Specifically, the mean number of detected BGCs per individual at each time point was 867 (visit 1), 871 (visit 2), 894 (visit 3), and 887 (visit 4). No significant differences were observed in the alpha-diversity distributions between visits (Figure S1a). About 74% of BGCs were consistently shared between consecutive visits within the same individual. Bray‒Curtis distance analyzes between the initial visit and subsequent visits indicated that the microbial BGC composition diverged progressively over time. While the BGC change was not significant at 6 months (Bray‒Curtis distance: visit 1 vs. visit 2 compared to visit 1 vs. visit 3, Mann‒Whitney U test, *p* = 0.138), it was statistically significant after 9 months (Bray‒Curtis distance: visit 1 vs. visit 2 compared to visit 1 vs. visit 4, Mann‒Whitney U test, *p* = 0.009) (**Figure S1b**). These results demonstrate that the BGC profile can change over longer timeframes, motivating an investigation into BGC establishment in the gut.

### Uncovering the mobility, resistance and pathogenicity of the BGC catalog

Integrating biological annotations into the analysis of BGCs enhances our understanding of their functional roles and evolutionary dynamics, facilitating the prioritization of promising BGCs for subsequent product characterization.^12^ Mobile genetic elements (MGEs) are known to drive evolutionary diversification in certain BGCs.^23^^,^^24^ To investigate the potential for MGEs within the BGC catalog, we searched for recombinase markers indicative of MGEs (Methods).^25^ In total, 9.1% of the BGCs contained recombinase genes, with transposases identified as the most common recombinase type (Figure 1d). Certain SM exhibit antimicrobial activity and consequently, their biosynthesis often requires the presence of resistance mechanisms within the microbes to ensure self-protection.^26^ To evaluate this possibility in the BGC catalog, we used DeepARG,^27^ to predict antibiotic resistance genes (ARGs). A total of 20.8% of the identified BGCs contained at least one predicted ARG, with *multidrug resistance* representing the most frequent predicted resistance type (Figure 1e). Bacterial pathogens commonly harbor virulence factors (VFs), which confer the ability to invade, colonize, survive within the host, and potentially cause host damage.^28^ Screening of our BGC catalog against the Virulence Factor Database VFDB^29^ demonstrated that 15.4% of the BGCs identified in the Wellness cohort contained genes homologous to known VFs. The most frequently detected VF category belonged to immune modulation and was specifically represented by lipopolysaccharides (LPS) (Figure 1f, Table S1).

Genes involved in SM are typically not essential for bacterial survival. Thus, we explored the distribution of their pangenome annotations^30^^,^^31^ and observed the median fraction of accessory genes being larger than the median fraction of core genes (Figure S2). To determine whether predicted BGCs exhibit a similar abundance pattern to their host species, we calculated the Spearman correlation between the abundance of each BGC and the abundance of its corresponding metagenomic species pan-genomes (MSPs) (Figure 1g). About half of the BGCs displayed correlations below 0.6 with their assigned MSPs, suggesting that these BGCs are part of the accessory pangenome and are only present in a subset of strains within a species. We observed that BGCs predominantly composed of core genes (fraction of core genes in BGC > 0.7) show high correlation values with their assigned host taxonomy and were significantly depleted in recombinases (4.8-fold depletion; hypergeometric test, *p* = 1.4 × 10^−17^) (Figure S2a–c). Conversely, BGCs exhibiting low correlation values with their assigned host taxonomy (Spearman correlation, r < 0.3) showed significant recombinase enrichment (1.45-fold enrichment; hypergeometric test, *p* = 0.0003) (Figure S2d).

### Transient and persistent BGCs in the healthy human gut microbiome and their functional differences

**Figure 2.**
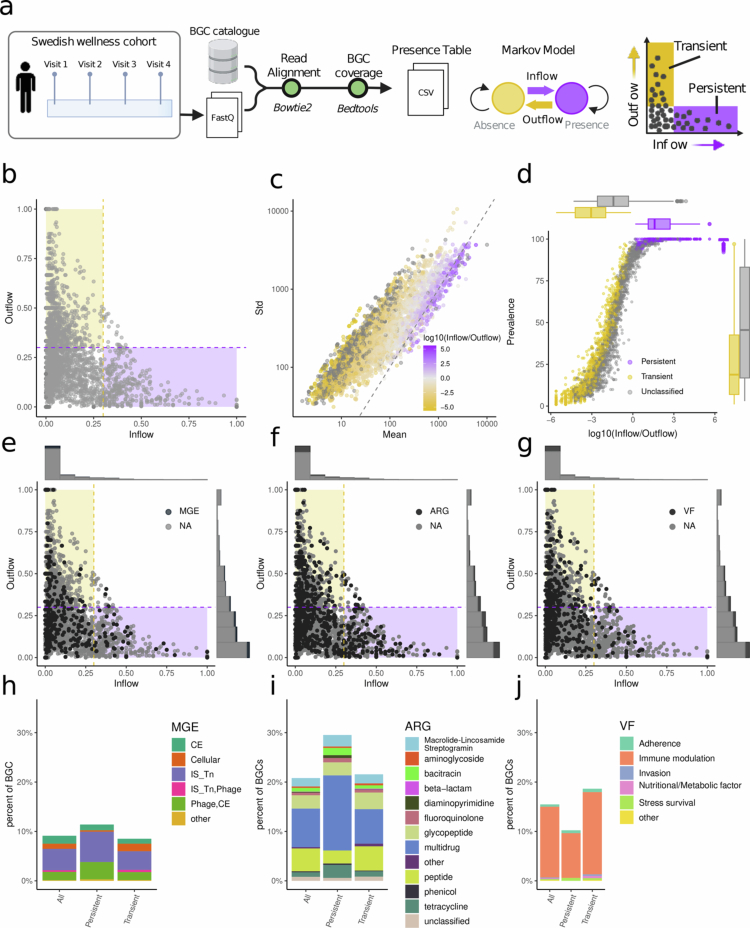
Transient and persistent BGCs in the wellness cohort. a) Scheme showing the workflow for the analysis of longitudinal data in the Wellness Cohort. The shotgun metagenomic samples were mapped against the BGC catalog to construct a presence table, which was used to fit a 2-state Markov model for each BGC and to estimate inflow and outflow probabilities. This approach enabled the identification of transient and persistent BGCs within the catalog. b) Scatterplot of the estimated inflow and outflow probabilities for each BGC in the catalog. c) The mean and standard deviation of the relative abundance values of the BGCs within the Wellness Cohort. The dashed line denotes identity. The color scale shows the inflow to outflow ratio for each BGC. The gray color denotes the NA values. d) The scatter plot shows the relationship between the log10 (inflow/outflow) values and the prevalence (percent of subjects where the BGC was detected at least once during the study length) in the subjects within the Wellness Cohort. Scatter plots of the inflow and outflow values of each BGC labeled by the presence of e) mobile genetic elements (MGE) (CE, conjugative elements; IS, insertion sequences; Tn, transposons), f) antibiotic resistance genes (ARGs), and g) virulence factors (FFs) within the BGC. Bar plots of the relative abundance of BGCs in the catalog associated with h) mobile genetic elements, i) antibiotic resistance genes, and j) virulence factors.

Thus far our results revealed a progressive shift in the BGC profile among subjects across time points. Next, we model the probability of gaining (inflow) or losing (outflow) BGCs over time. The raw metagenome data were mapped against the BGC catalog, and the coverage was calculated. We then fitted the Markov model to the longitudinal presence or absence of BGCs obtained from each subject in the Wellness cohort (Figure 2a**,** Figure S3a, Methods).^18^ A high outflow probability means that if a BGC is present, it is likely to be lost in the following visits. Conversely, a low outflow probability suggests that the BGC tends to persist over time. Similarly, a high inflow probability indicates that if a BGC is absent, it has a good chance of appearing in subsequent visits, while low inflow probability suggests that it is likely to remain absent. Based on the estimated inflow and outflow probabilities (Figure 2b, Table S1), we defined two distinct groups of BGCs: transient (tBGC) and persistent (pBGC). The transient group includes BGCs with high outflow probabilities (>0.3) and low inflow probabilities (<0.3), indicating frequent loss and limited reacquisition. In contrast, the persistent group consists of BGCs with high inflow probabilities (>0.3) and low outflow probabilities (<0.3), suggesting long-term persistence across time points. As expected, most pBGCs were associated with species from the *Clostridia* class, followed by *Bacteroides*. A similar trend was observed for tBGCs, where *Clostridia* remained the most abundant class, followed again by *Bacteroides*. However, *Gammaproteobacteria* and *Bacilli* were more frequently observed among tBGCs than in the persistent group (Figure S3b). Notably, BGCs with *Bacteroides ovatus* taxonomic annotations were found in both the transient and persistent groups, suggesting that specific strains within the same species may differ in their capacity to colonize and persist in the human gut. This highlights that BGC stability over time is not solely determined by species identity.

Moreover, we observed a strong positive correlation between the mean relative abundance and the standard deviation across BGCs, indicating that clusters with higher average abundance tend to exhibit greater variability (Figure 2c). Additionally, as the standard deviation increased relative to the mean, the inflow-to-outflow ratio decreased (Figure 2c). We observed a strong association between the 1-y prevalence of each BGC, defined as the percentage of individuals in which a BGC was detected at least once during the sampling period, and its inflow-to-outflow ratio (Figure 2d). pBGCs were found at least once in nearly all the subjects over the year, indicating that if a pBGC falls below the detection limit at one time point, it is likely to reappear in subsequent visits. In contrast, tBGCs displayed a wider range of prevalence values, consistent with limited acquisition and reduced stability over time.

We compared BGC-estimated inflow and outflow values across two independent longitudinal cohorts (the American HPFS cohort PRJNA354235, and the Italian DINAMIC cohort PRJEB33500).^32^^,^^33^ Despite differences in cohorts (such as geography or sampling timepoints) these values were significantly correlated between cohorts, suggesting they reflect at some extent true biological features of BGC turnover in the gut microbiome (Figure S4).

To investigate potential functional differences between pBGCs and tBGCs, we assessed whether these groups were enriched or depleted in specific genetic features, including ARGs, MGE markers, and VFs. These features could help revealing the types of biological functions encoded by each BGC group in the gut microbiome. ARGs were significantly overrepresented among pBGCs, with a 1.4-fold enrichment (hypergeometric test, *p* = 4.2 × 10⁻⁵). In contrast, VFs were significantly underrepresented in pBGCs, showing a 1.46-fold depletion (hypergeometric test, *p* = 0.005). No significant enrichment or depletion of MGE markers was observed in either pBGC or tBGC groups (Figure 2e–j). These results suggest that pBGCs are more frequently associated with functions related to competitive maintenance within the microbiome, while tBGCs may include functions more closely linked to opportunistic interactions with the host.

### pBGCs showed higher colonization rates in fecal microbiota transplantation

In microbial communities, the repertoire of BGCs plays a central role in shaping the profile of SM,^19^ which in turn mediates interspecies interactions, competitive dynamics, and host modulation. Introducing an exogenous community of microbial species and strains, such as through FMT, induces a substantial shift not only in taxonomic composition but also in the biosynthetic potential of the recipient's microbiome, where these populations come into contact and compete for niche establishment. Consequently, FMT provides a unique opportunity to monitor how the SM potential changes with induced shifts in the microbiome community. Observing the functional differences between pBGCs and tBGCs, and considering the higher inflow probability of pBGCs in a complex and dynamic microbial community, we investigated the potentials of the colonization of the pBGCs and tBGCs transferred from healthy donors to recipient patients under FMT treatments.

**Figure 3.**
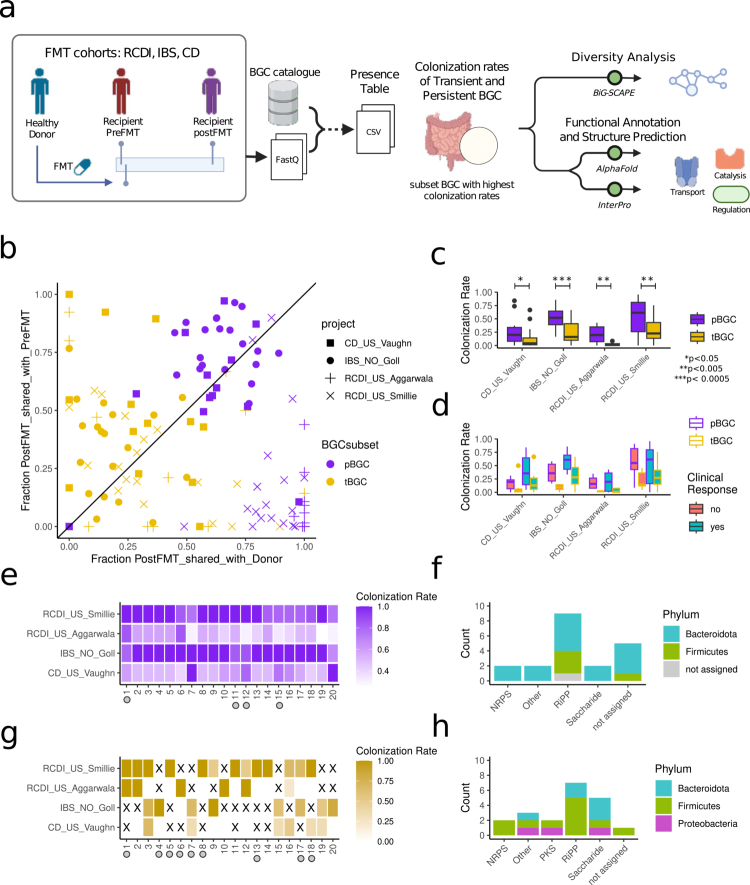
Persistent and transient BGC colonization during fecal microbiome transplants. a) Scheme showing the FMT analysis workflow. Shotgun metagenomic samples from four different FMT cohorts were mapped vs. the BGC healthy catalog to estimate the colonization rates of transient and persistent BGCs after FMT treatment. Diversity analysis, functional annotation, and structure prediction were performed on colonized pBGCs and tBGCs. b) Scatter plot of the fraction of tBGCs and pBGCs present in post-FMT recipient samples also present in donor samples (x-axis) vs. the fraction of BGC present in post-FMT recipient samples also present in pre-FMT recipient samples (y-axis). Each dot represents an FMT triad, measuring the shared fraction of BGCs. c) Boxplot of the colonization rates (total number of BGC present in donor sample and post-FMT recipient sample but not in pre-FMT recipient sample, divided by the total number of BGCs present in the donor but absent in the pre-FMT recipient) of the FMT triad in the pBGC and tBGC groups. d) Box plot of the colonization rates of the FMT triads grouped by reported clinical response in the persistent and transient BGC groups. e) Heat map of the top 20 pBGCs ranked by colonization rate. The gray circles at the bottom show BGCs with MultiGeneBlast hits against the MiBIG database. f) The different types of BGC in the top 20 pBGCs are colored by taxonomic phylum. g) Heat map of the top 20 tBGCs ranked by colonization rate. The gray circles at the bottom show BGCs with MultiGeneBlast hits against the MiBIG database, *X* denotes NA values. h) The types of BGC in the top 20 tBGCs colored by taxonomic phylum.

To do so, we analyzed FMT triads (donor, pre-FMT recipient and post-FMT recipient) in four publicly available metagenomic FMT datasets (Table S2).^34-37^ One from Norway involving patients with irritable bowel syndrome (IBS, 20 triads),^36^ and three from the United States, one focused on Crohn's disease (CD, 13 triads)^37^ and two on recurrent *Clostridium difficile* infection (RCDI, 32 triads).^34^^,^^35^ Using our BGC catalog as a reference, we tracked the presence of these clusters across donor samples, as well as pre-FMT and post-FMT recipient samples (Figure 3a, Table S2). Among the 1303 BGCs classified as transient in the wellness catalog, 292 were detected in at least one of the FMT samples. In contrast, 340 out of the 342 BGCs classified as persistent were found in at least one FMT sample. These observations highlight the broader distribution and higher detectability of pBGCs across diverse populations.

Given that pBGCs show long-term stability in healthy individuals, we hypothesized that they would also exhibit higher colonization rates in FMT recipients with underlying dysbiosis compared to tBGCs. The mean value of the shared fraction of pBGCs in post-FMT recipients and also present in donors was 76% (S.D. 20%). In contrast, the mean value of the shared fractions of tBGC in post-FMT samples also present in donors was 28% (S.D. 23%). We also observed the mean fraction of pBGC present in post-FMT and also present in pre-FMT samples in RCDI samples was 15.1% (S.D. 22%), whereas for non-RCDI samples the corresponding value was 69.9% (S.D. 21%). This clear distinction in pBGCs is not as marked in the tBGC subset where the mean values of the fraction in the post-FMT shared in pre-FMT were 29% (S.D. 25%) and 41% (S.D. 27%), for RCDI and non-RCDI, respectively (Figure 3b, Figure S5a).

We then analyzed colonization rates in FMT triads for both tBGC and pBGC groups. Across cohorts, pBGCs displayed higher colonization rates (Figure 3c). Although colonization rates were generally lower for tBGCs, in a small number of patients tBGC colonization exceeded that of pBGC (Figure S5b). Projecting the clinical responses of the patients based on tBGC and pBGC, we also observed a trend indicating that higher colonization rates (both in pBGC and tBGCs) were more frequently observed in cases with positive clinical responses (Figure 3d). To test the expression of pBGCs and tBGCs in the gut, we analyzed meta-transcriptomics data from stool samples before and after FMT treatment in a case study of chronic antibiotic-resistant pouchitis.^38^ Seven samples from two different healthy donors and 42 samples from three different pouchitis patients were analyzed (Table S3). We observed a higher expression of pBGC compared to tBGCs in healthy donor samples (Figure S6 a, c). Two of the three patients before FMT have similar expressions of pBGC and tBGCs, the third patient shows higher expression of pBGCs compared to that of tBGCs. All patients received antibiotics before the first FMT resulting in a decrease in expression of both pBGCs and tBGCs. In the immediately following days we observe increased expression of pBGCs compared to tBGCs (Figure S6 b, d).

### Functional impact of high-colonizing pBGCs on host physiology

Among the top 20 BGCs with the highest colonization rates within tBGCs and pBGCs, we found that Bacteroidota was the most represented phylum in the pBGC group, followed by Firmicutes (Figure 3e and f). In contrast, in the tBGC group, Firmicutes was most prevalent, followed by Bacteroidota and Proteobacteria (Figure 3g and h). The species associated with the highest-colonizing pBGCs included *Bacteroides ovatus*, *Bacteroides uniformis*, *Bacteroides vulgatus*, *Mediterraneibacter faecis*, *Bacteroides xylanisolvens*, *Blautia wexlerae*, and *Alistipes onderdonkii*. Species linked to top-colonizing tBGCs included unclassified *Blautia*, *Clostridia bacterium UC5.1-1D10*, *Escherichia coli*, *Agathobacter rectale*, *Erysipelatoclostridium ramosum*, *Ruthenibacterium lactatiformans*, *Anaeromassilibacillus sp. An250*, unclassified *Hungatella*, *Roseburia intestinalis*, *Clostridium innocuum*, *Bacteroides nordii*, *Butyricimonas virosa*, and *Dorea sp. CAG:317* (Table S4, Figure S5c, d). Among the top 20 tBGCs and pBGC with the highest colonization rates, we observed that the most frequent BGC types included NRPS, PKS, and RiPPs. Five pBGCs and six tBGCs were associated with predicted ARGs, while a smaller subset was linked to MGEs (one pBGC and two tBGCs). We also annotated the genes for the top 20 tBGC and pBGC using InterProScan^39^^,^^40^ for the gene ontology (GO) terms, among them, the molecular functions *glycosyltransferase activity* and *ATP binding*, as well as the biological processes *transmembrane transport* and *fatty acid biosynthetic process*, exhibited the most pronounced differences, appearing more frequently in the top 20 tBGCs (Methods, Figure S7, Table S5). The molecular function protein binding and the biological process carbohydrate metabolic process was more frequently observed in the top 20 pBGCs.

To further investigate the functional potential of these BGCs, we searched for sequence homology against the Minimum Information about a Biosynthetic Gene cluster (MIBiG) database.^41^^,^^42^ Four of the top 20 pBGCs had at least one gene showing homology to BGC genes in MIBiG. pBGC-12, an arylpolyene**-**type BGC from *Bacteroides xylanisolvens* with 74.1% mean colonization rate, showed good similarity and synteny conservation (MultiGeneBlast score = 0.29) to the flexirubin BGC from *Chitinophaga pinensis* (Figure S8).^43^^,^^44^ Flexirubin is a characteristic pigment of the *Bacteroides* group with antioxidant properties and potential therapeutic applications in free radical-related diseases,^45^ offering protection against hepatotoxins.^46^

There were other pBGC with lower similarity scores (MultiGene Blast score < 0.16). pBGC**-**11, with 74.2% mean colonization rate (RiPP**-**type BGC from *Bacteroides ovatus*), which contains one gene sharing sequence similarity with a core biosynthetic gene from the thuricin CD *α* cluster of *Bacillus thuringiensis* (BGC0000599), a post-translationally modified bacteriocin with narrow-spectrum activity against *C. difficile.*^47^ Similarly, another RiPP-type BGC from *B. uniformis* (pBGC-15 with a 72% mean colonization rate) shared homology with the CirD gene from the circularin A cluster of *Clostridium beijerinckii* (BGC0000488), which is known for its antimicrobial activity against a broad range of Gram-positive bacteria.^48^

We also identified nine BGCs within the top 20 tBGCs where at least one of their constituent genes showed sequence similarity to entries in the MiBIG database. Among them, tBGC-4 and tBGC-7 are associated with *E. coli* and have been previously experimentally characterized. tBGC-4 synthetizes Aryl polyene, a metabolite that increases protection of *E. coli* from oxidative stress and contributes to biofilm formation.^49^ tBGC-7 is a PKS**-**NRPS BGC that codes for the biosynthetic pathway of yersiniabactin (MIBiG id BGC0001055). Yersiniabactin is a siderophore essential for virulence in *Yersinia* species, playing a critical role in bubonic and pneumonic plague.^50^ In *E. coli*, this cluster is located within a pathogenicity island, adjacent to the colibactin gene cluster, a genotoxin shown to induce DNA damage in host cells and linked to colorectal cancer.^16^ It should be noted that this BGC was generally undetectable or present at low levels in most individuals within the Wellness cohort but exhibited sporadic and sharp increases in a small subset of subjects over the course of the year (Figure S9).

### Predicted linear azol(in)e-containing peptides for a pBGC from *Bacteroides ovatus*

**Figure 4.**
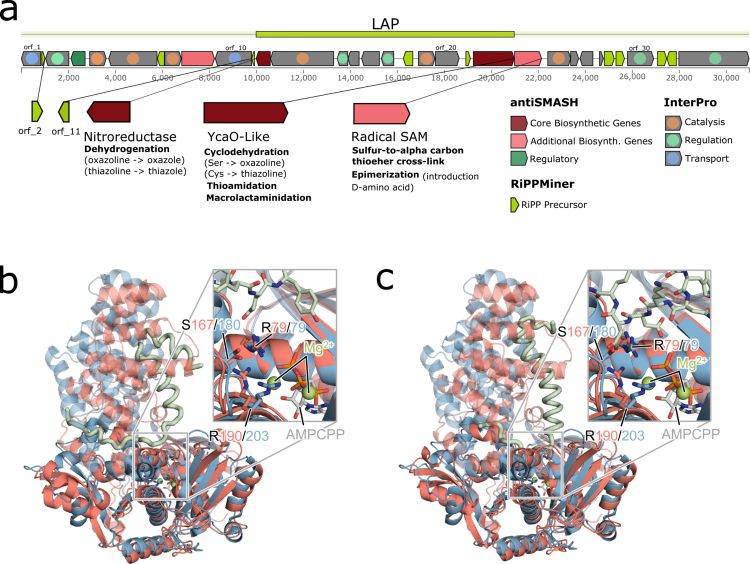
Functional annotations and structural analyzes of pBGC-11. a) ORFs organization within the pBGC-11 annotated by antiSMASH, RiPPMiner and manual classifications based on InterPro outputs. Structural alignments of the multimeric predictions of ORF22 (salmon) in complex with the predicted RiPP precursors (light green) b) ORF2 and **c)** ORF11 onto the crystal structure of YcaO from *E. coli* (blue; PBD code 4Q85). The Cα alignment (5 cycles, 2 Å outlier cutoff rejection) was performed on the annotated YcaO cyclodehydratase domains (PFAM PF02624). The aligned Cα RMSDs were 1.8 Å and 1.6 Å for the ORF22/ORF2 and ORF22/ORF11 predictions, respectively. Unaligned regions are also shown (partially transparent). The Mg^2+^ ions and the non-hydrolysable ATP analog, AMPCPP, bound to the active site of the YcaO model are displayed. The magnification boxes focus on the YcaO domain active site consisting of conserved arginine residues and a serine, AMPCPP, and Mg^2^^+^ ions.

Among the 20 colonized pBGC, we selected as a proof of concept a cluster (pBGC-11) from *Bacteroides ovatus*, for further analysis due to one of its genes sharing sequence homology with a key biosynthetic gene from the thuricin CD *α* gene cluster, a RiPP with narrow-spectrum antimicrobial activity against *Clostridioides difficile*.^47^ This connection is in our opinion particularly significant given that two of the FMT cohorts included in our study involved patients with recurrent *C. difficile* infection. Combining extensive functional annotation^40^ and using recent advances in protein structure prediction,^51^^,^^52^ we examined the gene composition of pBGC-11 to generate hypotheses about the potential synthesized molecule. Two open reading frames (ORF12 and ORF22) were predicted to encode enzymes involved in the biosynthesis of linear azol(in)e-containing peptides (LAPs),^53^^,^^54^ a RiPP subclass (Figure 4a). These enzymes modify serine, threonine and cysteine residues to form oxazoles and thiazoles. ORF23 showed strong sequence similarity to the SAM radical enzyme Thuricin C, part of the thuricin CD *α* cluster. The canonical CX₃CX₂C motif required for coordination of a [4Fe-4S]⁺ cluster is conserved in ORF23, supporting the potential for catalytic activity (Figure S10). Although nine of the ORFs in the cluster were predicted as RiPP-related by RiPPminer,^55^ none exhibited homology to the *α* or *β* peptides of thuricin CD. Additionally, pBGC-11 lacks an identifiable transporter gene (according to antiSMASH-provided annotations), a common feature in RiPP clusters (Figure 4a). Using InterPro annotations, we categorized genes within predicted BGCs into one of three functional groups: regulation, transport, or catalysis (Table S6). We found that ORF1 and ORF10 show transport functions and are located next to predicted RiPP precursors, making them possible candidates for transporting the hypothetical final product. Then, we conducted a prediction of paired multimer predictions between all eight predicted RiPP precursors and ORF 22, the first enzymatic step in LAP biosynthesis (Figure 4a), to propose plausible substrates for the enzyme. ORF 22 is predicted to fold in two domains, the *N*-terminal domain binding ATP, homologous to YcaO, and a C-terminal domain located on top of the active site, with a groove formed in between the two domains (Figure 4b and c). We predicted putative RiPPs coded by ORF 2 and ORF 11 fit within this groove (Figure 4b and c) in which the C-terminal domain stabilizes the peptide precursor peptide, where the active site could catalyze the chemical transformation of the peptide(s).

### Biosynthetic diversity of BGCs transferred during FMT treatments

To explore the SM diversity encoded by pBGCs and tBGCs transferred during FMT, we focused on the subset of clusters annotated using antiSMASH. Unlike tools such as DeepBGC and SanntiS, which apply deep learning models to predict novel or atypical BGCs, antiSMASH relies on a rule-based strategy. Although more conservative, this approach provides richer functional annotations by linking predicted BGCs to known biosynthetic types. From the Wellness BGCs catalog, we identified 259 antiSMASH-annotated BGCs involved in FMT colonization events, 141 classified as persistent and 118 as transient. These included 216 RiPPs, 22 NRPS, and 19 in other categories. To assess their biosynthetic diversity, we used BiG**-**SCAPE^56^ which constructs sequence similarity networks based on gene content, and groups BGCs into families. BiG-SCAPE also compares the input BGCs to those in the MIBiG database, providing hints on their function(s) based on gene similarity contents. The resulting sequence similarity network, constructed using a relaxed similarity threshold (cutoff = 0.8), revealed that most transferred BGCs did not match any known clusters in the MIBiG database, implying a high degree of unexplored SMs.

**Figure 5.**
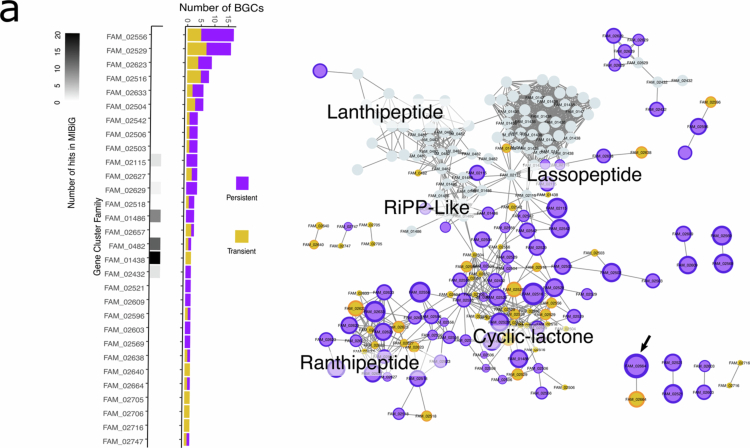
Biosynthetic diversity analysis of RiPPs in colonizing bacteria after FMT. Biosynthetic diversity analysis of pBGC and tBGCs predicted as RiPPs type by antiSMASH colonizing after FMT. The analysis was performed using BiG-SCAPE. Each node in the similarity network represents a BGC, and edges connect BGCs sharing similarity above the specified cutoff (see methods). Node labels indicate the assigned family after the clustering step is performed within the BiG-SCAPE pipeline. Node size and edge width are proportional to the mean colonization rate in the four FMT cohorts. The gray nodes represent BGCs from the MIBiG database. The black arrow in the network points to the BGC with the highest colonization rate in pBGC-1. The heat map (grayscale, left) shows the number of similar BGCs within the MIBiG database when grouped by gene cluster family. The bar plot shows the number of pBGCs and tBGCs within each family.

RiPPs displayed the greatest diversity (Figure 5). Among the BGC families containing at least two members, only six showed similarity to known BGCs. The remaining families were uncharacterized, including two large and distinct groups predicted to encode ranthipeptides and cyclic**-**lactone autoinducers. Cyclic lactones have been described as quorum-sensing signals and cross**-**inhibitors, modulating the expression of accessory functions in *Staphylococcus* species,^57^ while ranthipeptides in *Clostridium* are implicated in cell**–**cell signaling and metabolic regulation.^58^ Therefore, we propose that the regulation of microbial behavior represents a central ecological function of many RiPPs in the gut microbiome.

Notably, both pBGCs and tBGCs were found within the same biosynthetic families. This indicates that, despite differences in temporal stability, these clusters may share a common evolutionary origin, as suggested by their conserved gene contents. However, because families were defined using a relaxed similarity threshold, the grouping reflects general relatedness rather than strict functional equivalence. As a result, there is ample room for structural and functional divergence among BGCs within the same family, potentially leading to differences in the SM and their roles.

## Discussion

Tracking microbial communities over time has uncovered patterns of stability and change, showing how shifts in microbial composition can influence disease susceptibility.^18^^,^^59^ Such a dynamic understanding is crucial for translating basic microbiome science into viable therapies. Our study turns the focus from taxonomic composition to the potential of SM in the microbiome and the impact on host‒microbiome interactions. Direct analysis of BGCs offers a path to link microbial products to the molecular mechanisms driving their effects. The reconstruction of a functional catalog of gut-associated BGCs in healthy individuals and defining pBGC and tBGC provide a novel feature of SM in the establishment of a new microbial community structure. Groups of pBGCs that have enrichment for antibiotic resistance genes and depletion for virulence factors, suggest they may encode functions beneficial to the host, potentially contributing to microbiome stability and resilience. Assessing the behavior of these BGCs during FMT revealed that pBGCs are more likely to be successfully engrafted and maintained in recipient microbiomes. These findings provide insights into 1) the stability and distribution of SM potential in the healthy population, 2) the association of persistence in time of BGCs to their roles in the host, and 3) the transmission and expression of BGC within FMT.

### pBGC and tBGC colonization and relation to fitness and virulence in FMT

Strong evidence support SM can mediate host-colonization.^60-62^ Here, we observed that pBGCs exhibited higher colonization rates and expression compared to tBGCs during FMT. The functions linked to the pBGC suggest they provide benefits to the host,^43-48^ potentially contributing to microbiome stability and resilience. Enrichment of antibiotic-resistant genes, particularly multidrug efflux pumps, in pBGCs suggests enrichment of antibiotic activity of the SM product,^12^ possibly conferring a competitive advantage for microbes with these BGCs^63^ by displacing susceptible microbes in the recipient gut during FMT. In contrast, VFs are more common in tBGCs in relation to pBGCs, aligning with the observation that transient species are more likely to include opportunistic pathogens.^18^ The set of tBGCs may confer short-term adaptive benefits to species poorly suited for stable colonization at the expense of the host health. For example, the tBGC responsible for the synthesis of yersiniabactin (a siderophore linked to virulence in both *E. coli* and *Yersinia* species)^50^ showed sharp temporary enrichment followed by rapid depletion in a few subset of the subjects within the healthy cohort. The presence of VFs in tBGCs may support opportunistic invasion strategies allowing establishment in a perturbed environment.

### Prioritization for functional characterization and potential for therapeutic applications

Only a small fraction of gut microbiome BGCs have been functionally characterized.^14^^,^^64^ Experimental characterization of BGCs is a resource-demanding process,^65^ making prioritization a key step for making this process more effective.^12^ By linking longitudinal BGC dynamics to colonization success, our work highlights candidate BGCs for downstream characterization, with potential implications in microbiome-based therapies. The classification of BGCs into persistent and transient groups sets a framework for the exploration of therapeutic applications. The ubiquity and safety profile implied by their persistence in addition to potential beneficial effects of pBGCs and associated SM^43-48^ suggest they are well tolerated and possibly coadapted to human physiology, making them attractive candidates for drug development. As an illustrative example, pBGC-12 showed high similarity to gene clusters known to synthesize flexirubin. This SM have been shown to exhibit broad pharmacological properties, including antimicrobial,^66^ anticancer,^67^ antioxidant,^45^^,^^68^ and protective effects against carbon tetrachloride-induced liver injury,^46^ safety of flexirubin extracts also has been confirmed.^69^ The tBGCs, particularly those associated with VFs, may represent strategic targets for antivirulence therapies. Targeting either the molecules involved in host invasion or the genes responsible for their synthesis could disarm pathogens while potentially reducing resistance development^70^ and preserving the gut microbiota.^71^ In *Yersinia pestis*, the loss of yersiniabactin uptake or yersiniabacin synthesis results in a nearly complete inability to cause fatal bubonic disease.^50^^,^^72^ FMT is generally safe and effective for several conditions, but it is important to carefully assess the risks and benefits for each patient and to monitor for any side effects before and after the procedure.^73^^,^^74^

Despite recent advances, there is a limited understanding of the functional microbial contributors to successful clinical outcomes.^75^ In addition to antimicrobial activity, it would be plausible as well to find some of the products of the BGCs identified could have a role in the host immune modulation. This idea is supported by previous studies showing that secondary metabolites derived from bacterial sources can exert immunomodulatory effects.^76^ In line with this, most gene annotations linked to virulence factors in our BGCs fall within the immune modulation category.

We identified a fair amount of RiPPs with putative regulatory functions, including quorum sensing and interspecies communication.^57^^,^^58^ Several RiPPS subclasses derived from the human microbiota have been characterized before, among them we found Lanthipeptides,^77^ Sactipeptides,^78^ and Microcins.^79^^,^^80^ Besides antimicrobial activity found in experimentally characterized RiPPs, other interesting biological properties have been reported, such as pro-apoptotic activity^79^ and regulatory roles inside the bacterial cells.^80^ Among the RiPPs here, several were related to cyclic-lactones and ranthipeptides, subclasses that have also been linked to regulatory functions. We anticipate that further characterization of them may reveal novel mechanisms to modulate gut microbial helping to better understand FMT outcomes and to get a deeper understanding of the interactions in the human gut microbiome.

### Study limitations

BGCs are embedded within the genomes of bacterial species; therefore, their temporal dynamics largely reflect the population dynamics of their host strains. BGC-encoded natural products can confer additional traits that extend beyond core metabolic functions, potentially influencing strain fitness, persistence, and colonization within the host. Variation in BGC content among strains of the same species may therefore contribute to differences in persistence, although disentangling BGC-specific effects from broader strain-level turnover remains an area for future investigation. While our findings BGC dynamics in FMT remain correlative, they could be further validated experimentally by testing the effects of strains lacking such BGCs on gut colonization and prevalence, as well as functionally characterizing their biosynthetic products. The absence of comprehensive metadata on factors known to influence microbiome, including antibiotic exposure, diet, and host genetics, limit the analysis from fully accounting for these potential confounding variables. Future studies incorporating detailed longitudinal metadata on host and environmental factors will be essential to disentangle their respective contributions to BGC dynamics.

## Materials and Methods

### Developing Nextflow pipeline to create BGC catalog and abundance estimation using the shotgun metagenomic data

The pipeline for BGC prediction and abundance estimation consists of two main steps: BGC reference database creation and BGC abundance estimation using the metagenomic samples. For the first stage, SPADES was used for assembly of shotgun metagenomic paired-end sequences for each sample with the option -meta and default parameters.^22^ After assembly, BGC prediction was performed on the contigs larger than 5kbp using antiSMASH (using the following options --cb-general, --cb-knownclusters, --asf, --genefinding-tool prodigal-m),^10^ SanntiS,^9^ DeepBGC (using --prodigal-meta-mode),^3^ and GECCO (using –threshold 0.95)^5^ using default parameters. Sequence similarity between the predicted BGC from all samples was estimated using the mash software.^81^ The mash output was represented as a network, where BGCs were represented as nodes and one edge connected those BGC with a mash cutoff of sequence similarity above 0.95. The mcl software^82^ was employed for clustering of the resulting network and the longest sequence within each cluster was chosen as the representative BGC. For mapping metagenome data for each sample to estimate the BGC abundance, the resulting catalog of dereplicated BGC predicted on metagenomic assemblies, was used as input for building a bowtie2 index.^83^ Shotgun paired-end sequences form Wellness Cohort samples were aligned against the predicted BGCs and the best hits were retained for relative abundance estimation employing the RPKM formula. The pipeline was written with the Nextflow programming language^84^ and is available at GitHub (data availability).

### Alpha and beta diversity

The BGC relative abundance RPKM table was employed for the estimation of the Shannon alpha diversity index. The richness index is the count of present BGC within one sample, the BGC presence/absence matrix was employed for this estimation. The Bray‒Curtis distance was estimated using the RPKM information as input, and the Jaccard index was estimated using the presence/absence table as input. Alpha and beta diversity were calculated using the Python package scikit-bio.

### In silico mock community

For the simulation of the in silico mock community we employed CAMISIM^85^ pipeline. One hundred samples were simulated. First, a list of 100 genomes were randomly selected from the collection of bacterial genomes from the human gut.^86^ Using a binomial distribution and a parameter *p* = 0.7 the presence and absence of each genome was randomly generated. Then, the relative abundance for the present genomes was simulated using a log-normal distribution (*μ* = 0, *σ* = 1). The simulated relative abundance table and the complete genomes were employed as inputs for CAMISIM to emulate the shotgun sequencing reads at two different sequencing depths.

### Parameter optimization for BGC presence and absence detection using in silico mock community

To optimize the cutoff values for coverage fraction for considering whether a gene is present, and the fraction of genes present for considering whether a BGC present, we built a reference BGC database of the mock community, using the antiSMASH predicted BGC from the 100 genomes included in the in silico mock community. Then, we applied the Nextflow BGC prediction pipeline using as input the simulated fastq reads from the *in silico* mock community, and estimated recall and precision employing a grid search by varying both (gene-coverage and BGC-coverage) cutoff values. The selected parameters were 0.5 for BGC coverage (50% of the genes within a BGC needed to be detected to consider the BGC was present) and 0.3 for gene coverage (30% of the gene length had to be covered in the BAM alignments to consider the gene was present) (Figure S11).

### Markov chain model parameter estimation

Presence–absence profiles were fitted into a two-state Markov chain model (i.e. states of presence and absence) to estimate state transition probabilities between presence and absence of each BGC (R Markov chain package version: 0.10.0). We estimated the inflow probability of the state transition from absence to presence, and outflow probability of state transition from presence to absence. Based on estimated inflow and outflow probabilities we defined persistent (*P*_inflow_ > 0.3 and *P*_outflow_ < 0.3) and transient BGCs (*P*_inflow_ < 0.3 and *P*_outflow_ > 0.3) categories as previously reported.^18^

### BGC taxonomy annotation and pan-genome analysis

Each gene within a BGC was aligned to the IGC2 catalog using BLASTn^87^ (E-value ≤ 1e−10) to identify the closest matching reference gene and retrieve its associated taxonomy and pangenome category (core, accessory, or shared). This BLAST-based mapping anchors BGC genes to a curated human gut gene catalog, enabling aggregation and interpretation at the metagenome species pangenome level (MSP). We have added BLAST results for each gene within each BGC to facilitate assessment of assignment confidence, enabling a clear distinction between near-identical matches and weaker or ambiguous similarities (Table S7). IGC2 and MSP pangenome annotations provide additional biological context, specifically, information on whether genes belong to core, shared, or accessory components of an MSP pangenome offers insight into their evolutionary stability and likelihood of being detected across multiple species.^30^ Additionally, we performed taxonomic annotations of each BGC in the catalog using CAT software^88^ and included the annotations in the supplementary information (Table S8).

### Functional annotations in predicted BGCs

We performed a MultiGeneBlast (v1.1.14)^89^ search of each BGC in the catalog against the MIBiG database v4,^41^^,^^42^ using an identity cutoff of 30% and default parameters, annotations are provided in the supplementary material (Table S9). To predict the antibiotic resistance genes, the fasta sequence of each gene in the set of representative BGCs were annotated using DeepARG software^27^ with default parameters. Detection of recombinases associated with mobile genetic elements was performed using HMMer models reported in^25^ using a e-value cutoff of 1e-18. The hit with the lowest E-value was employed as the annotation label. A Blastx search of the BGCs nucleotide sequences was performed against the Virulence Factor Database^29^ using a e-cutoff value of 1e-10 and a lower percent identity cutoff of 60%. For BGCs with more than one hit, the hit with the highest sequence identity was selected for assigning the virulence factor annotation. InterProScan 5.71-102.0^39^^,^^40^ was used to annotate all CDSs in the predicted top colonizing BGCs, using default parameters. Each CDS was then manually assigned to a class: catalysis, transport, regulation, or unclassified. A non-repeating set of GO annotations for each CDS was also extracted from the InterProScan output. Precursor peptide prediction was performed using RiPPMiner tool with default parameters.^55^

### Gene cluster families, BiG-SCAPE, network representation of predicted BGCs

The set of representative BGCs predicted with antiSMASH were employed as input for BiG-SCAPE software^56^ employing three different cutoffs (0.3, 0.6, and 0.8). The networks were displayed using the Cytoscape software.^90^

### FMT colonization and engraftment

The colonization rate for a BGC was measured as the sum of BGC triads where the BGC was present both in the donor and post-FMT samples (but absent in pre-FMT) divided by the sum of triads where the BGC was present in donor and absent in recipient.

### Protein structure prediction and multimer structure predictions

The monomeric 3D structure of each sequence was predicted using AlphaFold 2.3.2^51^ on the Tetralith cluster (NSC, Sweden) using default parameters. Based on the annotations, structural predictions, and results from RiPPMiner, multimer predictions of RiPP precursors and annotated BGC-associated enzymes were generated with AlphaFold 2.3.2 in multimer mode.^52^

### Statistical tests and statistical metrics

The hypergeometric test, chi-square test, and Mann‒Whitney U tests were performed using R software. The colonization rate for an FMT triad (pre-FMT, donor, and post-FMT samples) was measured as the sum of BGCs present both in the donor and post-FMT sample (absent in recipient pre-FMT), divided by the sum of BGCs present in donor and absent in recipient. The BGC prevalence over 1-y was measured as the fraction of subjects where a BGC was predicted to be present in at least one of the four visits.

## Supplementary Material

Guevara_etal_Suppmaterial_2026_01_22.pdf Guevara_etal_Suppmaterial_2026_01_22.pdf

Guevara_etal_Suppmaterial_2026_02_20_Updated.docx Guevara_etal_Suppmaterial_2026_02_20_Updated.docx

## Data Availability

The Nexflow pipelines for BGC prediction are available at https://github.com/sysbiomelab/nf-BGCpred.git. The Nextflow pipeline for building the representative catalog and abundance estimation is available at https://github.com/sysbiomelab/nf-BGC.git. The Datasets employed can found under the following ENA data accession numbers: Swedish wellness cohort, PRJEB38984; RCDI_US_Aggarwala, PRJNA637878; RCDI_US_Smillie PRJEB23524; CD_US_Vaughn, PRJNA321058; IBS_NO_Goll, PRJEB36140; RNAseq Pouchitis cohort, PRJEB48996; the American HPFS cohort, PRJNA354235; and the Italian DINAMIC cohort, PRJEB33500.
