# Supplementary material for: Temporal dynamics of gut biosynthetic gene clusters link persistent colonization and engraftment in fecal microbiota transplantation
Source: Gut Microbes. 2026 Feb 25;18(1):2634469. doi: 10.1080/19490976.2026.2634469 (PMC12940132; doi:10.1080/19490976.2026.2634469)
Supplement: Guevara_etal_Suppmaterial_2026_02_20_Updated.docx [file KGMI_A_2634469_SM6266.docx]

# **Temporal dynamics of gut biosynthetic gene clusters link persistent colonization and engraftment in fecal microbiota transplantation**

# Fernando Garcia-Guevara^a^, Tom Resink^b,c^, Frederick Clasen^a^, Mathias Uhlén^d^, Adnane Achour^b,c^ and Saeed Shoaie^a,e^

# ^a^ Centre for Host-Microbiome Interactions, Faculty of Dentistry, Oral & Craniofacial Sciences, King's College London, UK

# ^b^ Science for Life Laboratory, Department of Medicine, Solna, Karolinska Institute, Solna, Sweden

# ^c^ Division of Infectious Diseases, Karolinska University Hospital, Stockholm, Sweden

# ^d^ Science for Life Laboratory, Department of Protein Science, KTH-Royal Institute of Technology, Stockholm, Sweden

# ^e^ Quantitative Systems Biology, Faculty of Medicine, Biruni University, Istanbul, Türkiye

# Corresponding author: saeed.shoaie@kcl.ac.uk

**

**Figure S1**. **BGC diversity** **Alpha and Beta diversity of the BGC content within samples**

**a)** The plot shows the Shannon index estimated for the subjects in the different visits. The violin plots show the distribution of the Shannon index values for each visit. Each dot represents one subject in a particular visit and the gray lines connect the same subject in different visits. **b)** the beta Bray-Curtis index values of samples compared to the initial visit of the same subject. The violin plots show the distribution of the Bray-Curtis index values. Each dot represents the beta diversity between a pair of samples from the same individual and the initial visit. The gray lines connect the dots from the same subject.

**Figure S2. Mobile Genetic Element presence in BGCs and pan-genomic annotations**

The histogram plots in a, b and c show the number of BGC with identified MGE in the catalogue in relation to the composition of their gene’s pan-genomic annotations. Pan-genomic annotations of the genes in the BGCs were extracted from the Integrated Gene Catalogue2 (IGC2 ^1^). **a)** The histogram shows the number of BGC in the catalogue vs the fraction of genes within the BGC annotated as core genes, the blue dotted line indicates the fraction of core genes equals to 0.7 **b)** The histogram shows the number of BGC in the catalogue vs the fraction of genes within the BGC annotated as shared genes. **c)**. The histogram shows the number of BGC in the catalogue vs the fraction of genes within the BGC annotated as accessory genes **d)** Histogram of Spearman correlation values between BGC abundance and MSP abundance sharing the same taxonomic annotation as the BGC; the blue dotted line indicates correlation equals to 0.3. The red dotted lines show the median values of the distributions. The blue rectangles outline the group of BGCs significatively enriched or depleted in MGEs.

**

**Figure S3***.* **Inflow and outflow values of BGCs based on the Wellness cohort**

**a)** The scheme shows the process for fitting the Markov model where time series of the presence/absence values for a single BGC were used to get estimates of the associated inflow and outflow probabilities. This process was applied for each BGC in the catalogue. **b)** The scatter plots show the inflow vs outflow values of all BGC in catalogue, separated by taxonomy class level and colored by BGC type.

Figure S4

**Figure S4**. Correlation between estimated inflow and outflow values in Wellness dataset and 2 independent longitudinal health cohorts (the American HPFS cohort PRJNA354235, and the Italian DINAMIC cohort PRJEB33500). The upper row shows the correlations between inflow probabilities, and the row below shows the correlation between outflow probabilities.

**

**Figure S5**. **Persistent and Transient BGC colonization during FMT within triads.**

**a)** The scatter plot shows the fraction of BGC present in post-FMT recipient samples that is also present in donor samples (x-axis) vs the fraction of BGC present in post-FMT recipient samples also present in pre-FMT recipient samples (y-axis). Each dot represents a FMT triad measuring the shared fraction of BGCs in the persistent or the transient group. Gray lines link pBGC and tBGCs of the same triad. **b)** The boxplot shows the colonization rates (total number of BGC present in donor sample and post-FMT recipient sample but not in pre-FMT recipient sample, divided by the total number of BGCs present in donor but absent in pre-FMT recipient) of the FMT triads in persistent and tBGC groups. Black dotted lines link pBGCs and tBGCs from the same triad. **c)** The bar plot shows the different types of BGC in the top 20 pBGCs, colored by taxonomic genus. **d)** The bar plot shows the different types of BGC in the top 20 tBGCs colored by taxonomic genus.

**Figure S6. Expression of pBGCs and tBGCs in meta-transcriptomics data from FMT for treating pouchitis patients.** A) Boxplots showing the number of expressed pBGCs and tBGCs in healthy donor samples. B) Plot showing the number of expressed pBGCs and tBGCs in the three FMT recipients across different sample collection time points. Labels indicate the subjects’ identifiers at the time of FMT. C) Boxplots of RPKM values for the top 20 colonizing pBGCs and tBGCs in healthy donor samples. D) Plot of RPKM values for the top 20 colonizing pBGCs and tBGCs in the three FMT recipients across the different collection time points.

**Figure S7.** **Difference in Gene Ontology annotations between tBGCs and pBGCs**

Gene Ontology (GO) annotations difference (in number of genes) between the genes in the top20 pBGC and the top20 tBGC **a)** of GO Molecular Function annotations and **b)** GO Biological Process Annotations.

**

**Figure S8. pBGC-12 from Bacteroides xylanisolvens is involved in the biosynthesis of flexirubin. a)** The scheme above shows the MultiGeneBlast comparison between pBGC-12 from *Bacteroides xylanisolvens* (top), and BGC0000839, responsible for the synthesis of flexirubin in *Chitinophaga pinensis^2^*. **b)** Reported structure of flexirubin from *Chitinophaga filiformis* and *Chitinophaga pinensis*.

**Figure S9***.* **Case example of Transient and Persistent BGC abundance dynamics trough time in the Wellness cohort**. **a)** The plot displays relative abundance values (RPKM) through time in all individuals of the pBGC detected in this dataset coding for a NRPS in *Blautia wexlerae*, the closest hit in the MIBIG databases is a dipeptide aldehydes biosynthetic gene cluster from Ruminococcus sp. 5_1_39BFAA (MIBiG database id BGC0001575). **b)** The plot displays relative abundance values (RPKM) through time in all individuals of the tBGC Yerseniabactin cluster (MIBiG database id BGC0001055).

**Figure S10. Conserved CX₃CX₂C motif in ORF23 of p-BGC1. Left)** Structural alignment of ORF23 (green), annotated as a radical S-adenosylmethionine (SAM) domain enzyme, onto the crystal structure of the radical SAM domain protein CteB from *Acetivibrio* *thermocellus* (purple; PDB: 5WGG^3^). The C_α_ alignment (5 cycles, 2 Å outlier cutoff rejection, 0.9 Å RMSD) was performed on the annotated radical SAM domains (CDD: cd01335). Unaligned regions are also shown (partially transparent). **Right)** Structural comparison of the conserved CX_3_CX_2_C motif at the [4Fe-4S] cluster and SAM binding site of the ORF23 prediction and CteB. The [4Fe-4S] cluster and SAM molecule from the CteB model are shown.

**

**Figure S11. Parameter optimization for BGC presence and absence detection**

**a)** Scheme showing employed metrics to define whether a BGC was considered present or absent. Fraction of gene coverage was measured as the length covered by aligned reads in a gene divided by the gene length. A gene is considered as present if the gene coverage is greater than a selected cutoff (values from 0-1).

**b)** Fraction of BGC coverage is measured as the number of genes present divided by the total number of genes in the BGC. The BGC is considered as present if the BGC coverage is greater than a selected cutoff.

**c)** Precision vs recall of the BGC calling method with varying cutoff values of gene coverage and BGC coverage, at two different simulated sequencing depths (0.4X and 2X). Numeric labels within the plot display the fraction of gene coverage cutoff, with lines that connect increasing gene coverage cutoff values using the same BGC coverage cutoff value and sequencing depth.

In our detection strategy, a BGC is considered absent if an insufficient number of reads align to the genes comprising the cluster. This definition means that absence reflects both detection limits mixed with true biological absence—*i.e*. a BGC may still be present but falls below the threshold imposed by the sample's sequencing depth and pipeline parameters. To control for this, our pipeline allows for the adjustment of two key parameters: minimum *fraction gene coverage* and *fraction of BGC-wide coverage*. We observed that both parameters interact and display different behaviours under different sequencing depths. Both precision and recall decrease with lower sequencing depth (0.4X vs 2X sequencing depth). Increasing the *fraction of BGC coverage* decreases the recall, while the effect is more pronounced at lower sequencing depth. Decreasing the *fraction of BGC coverage* cutoff at a higher sequencing depth decreases precision. There are varying optimum values for the *fraction of gene coverage* at low sequencing depths, depending on the *fraction of BGC coverage* selected. With higher sequencing depth as the *fraction of gene coverage* decreases the precision decreases. The selected parameters were 0.5 for BGC coverage (50% of the genes within a BGC needed to be detected to consider the BGC as present) and 0.3 for gene coverage (30% of the gene length had to be covered in BAM alignments to consider the gene as present). However, other factors such as overall sequencing depth, or community composition can also significantly influence detection performance. For example, highly diverse or uneven communities may dilute sequencing efforts across many taxa, reducing the sensitivity for detecting BGCs present at low abundance. These considerations underscore the importance of interpreting absence calls with caution, especially in metagenomic datasets with variable sequencing quality or complexity.

**Supplementary Tables.**

Table S1. Inflow and Outflow estimated probabilities.

Table S2. FMT metadata of metagenomic samples

Table S3. FMT metadata of meta transcriptomic samples

Table S4. Colonization rates of the top 20 pBGCs and top 20 tBGC

Table S5. GO annotations top20 pBGC and top 20 tBGC

Table S6. Manual Annotations of top 20 pBGC and top 20 tBGC into *Catalysis, Regulation*, and *Transport* categories based on IntePro annotations.

Table S7. Blast results of Genes in BGC catalogue against the IGC2 database

Table S8. Taxonomy annotation of BGC catalogue using the CAT software

Table S9. MultiGeneBLAST results of BGC catalogue against the MIBIG database.

1 Wen, C. *et al.* Quantitative metagenomics reveals unique gut microbiome biomarkers in ankylosing spondylitis. *Genome Biology* **18**, 142-142 (2017). <https://doi.org/10.1186/s13059-017-1271-6>

2 Schöner, T. A., Fuchs, S. W., Schönau, C. & Bode, H. B. Initiation of the flexirubin biosynthesis in Chitinophaga pinensis. *Microbial Biotechnology* **7**, 232-241 (2014). <https://doi.org/10.1111/1751-7915.12110>

3 Grove, T. L. *et al.* Structural Insights into Thioether Bond Formation in the Biosynthesis of Sactipeptides. *J Am Chem Soc* **139**, 11734-11744 (2017). <https://doi.org/10.1021/jacs.7b01283>
